# Exosomes derived from human umbilical cord blood mesenchymal stem cells stimulate regenerative wound healing via transforming growth factor-β receptor inhibition

**DOI:** 10.1186/s13287-021-02517-0

**Published:** 2021-08-03

**Authors:** Yan Zhang, Yingjin Pan, Yanhong Liu, Xiheng Li, Liang Tang, Mengna Duan, Jiang Li, Guokun Zhang

**Affiliations:** 1 Hospital of Stomatology, Jilin University, 1500 Qinghua Rd., Changchun, Jilin, 130021 China; 2 Jilin Provincial Laboratory of Biomedical Engineering, Jilin University, Changchun, China; 3 Center of Prosthodontics and Oral Implantology, Foshan Stomatology Hospital, School of Stomatology and Medicine, Foshan University, Foshan, 528000 China; 4 Center for Reproductive Medicine, Center for Prenatal Diagnosis, First Hospital, Jilin University, Changchun, China; 5 Affiliated Stomatology Hospital of Guangzhou Medical University, 39 Huangsha Ave., Guangzhou, 510080 Guangdong China; 6 Institute of Antler Science and Product Technology, Changchun Sci-Tech University, 1345 Pudong Rd., Changchun, Jilin, 130600 China; 7 Institute of Special Animal and Plant Sciences, Chinese Academy of Agricultural Sciences (CAAS), 4899 Juye St., Changchun, Jilin, 130112 China

**Keywords:** Cord blood stem cell transplantation, Exosomes, MicroRNAs, Myofibroblasts, Regeneration, Transforming growth factor beta, Wound healing

## Abstract

**Background:**

Scar formation is a common consequence of skin wound healing, and no effective treatment exists. Umbilical cord blood mesenchymal stem cells (UCB-MSCs) can improve wound healing; however, the role of UCB-MSCs remains unclear and whether they can ameliorate scar formation has not been fully elucidated.

**Methods:**

To determine the function of UCB-MSCs, we examined and compared the therapeutic effects of UCB-MSCs and UCB-MSC-derived exosomes (UCB-MSC-exo) on skin healing in rats. Moreover, UCB-MSC-exo-specific miRNAs were identified and their effects in inhibiting the human dermal fibroblast (HDF) differentiation into myofibroblasts were investigated.

**Results:**

Both UCB-MSCs and UCB-MSC-exo accelerated wound closure; reduced scar formation; improved the regeneration of skin appendages, nerves, and vessels; and regulated the natural distribution of collagen fibers in wound healing. Additionally, UCB-MSC-exo suppressed the excessive formation of myofibroblasts and collagen I and increased the proliferation and migration of skin cells in vivo and in vitro. Functional analysis showed that UCB-MSC-derived miRNAs were closely related to the transforming growth factor-β (TGF-β) signaling pathway, which could induce myofibroblast differentiation. We identified abundant miRNAs that were highly expressed in UCB-MSC-exo. miR-21-5p and miR-125b-5p were predicted to contribute to TGF-β receptor type II (TGFBR2) and TGF-β receptor type I (TGFBR1) inhibition, respectively. Using miRNA mimics, we found that miR-21-5p and miR-125b-5p were critical for anti-myofibroblast differentiation in the TGF-β1-induced HDF.

**Conclusion:**

The effect of UCB-MSCs in stimulating regenerative wound healing might be achieved through exosomes, which can be, in part, through miR-21-5p- and miR-125b-5p-mediated TGF-β receptor inhibition, suggesting that UCB-MSC-exo might represent a novel strategy to prevent scar formation during wound healing.

**Supplementary Information:**

The online version contains supplementary material available at 10.1186/s13287-021-02517-0.

## Background

Scar formation is a general consequence of wound healing after skin injury in adults [1–3], leading to psychological disorders and physical deformities. As there has not been an effective treatment until now, preventing or reducing scar formation is a significant problem in regenerative esthetics that urgently needs to be addressed. Pathogenesis of scar formation is assumed to occur via the recruitment and maintained differentiation of myofibroblasts, leading to excessive deposition of connective tissue (mainly collagen) [3, 4]. The differentiation of dermal fibroblasts in situ is the primary source of myofibroblasts, usually initiated by the TGF-β signaling pathway [4, 5]. In the process of wound healing, activated fibroblast proliferation and macrophage infiltration produce excess TGF-β [4, 5]. TGF-β binds to the TGF-β receptor (TGFBR) of fibroblasts, thus activating downstream signaling of TGF-β to induce myofibroblast differentiation, which further leads to the expression and extension of collagen fibers [2, 3, 6, 7]. In this regard, interfering with the TGF-β signaling pathway might inhibit myofibroblast differentiation from reducing scar formation.

Of all available treatments for the anti-scar formation and pro-ideal regeneration of skin appendages, mesenchymal stem cell (MSC) infusion has been considered a promising alternative strategy in numerous trials and clinical practices in the past few decades [8–10]. MSCs have several advantages that are essential for tissue repair, including relatively easy expansion in vitro, migration to the injured site, and differentiation into specific cell types required for tissue repair [9, 11–13]. Among the primary MSC sources that might be used for wound healing and regeneration of injured skin are adult-derived MSCs [9], fetal-derived MSCs [14], embryonic stem cells (ESCs) [15], and induced pluripotent stem cells [16]. Human umbilical cord blood-derived MSCs (UCB-MSCs) have been used to improve wound healing, and it has been reported that they could accelerate wound closure of diabetic wounds and promote the expression of anti-scarring factors in mechanical wounds, as well as stimulate the rejuvenation of human skin [17–21]. Compared with adult-derived MSCs, UCB-MSCs exhibit apparent advantages, including better-documented self-renewal, more multipotent differentiation properties, and lower immunogenicity [22, 23]. Compared with ESCs, UCB-MSCs are more easily obtained, as there are fewer related ethical issues [23]. However, the definite role of UCB-MSCs in vivo and their repair mechanisms in alleviating scar formation have not yet been fully elucidated.

The effect of MSCs in stem cell-based therapy is mainly via its secretion of paracrine pro-regenerative factors, including exosomes [9, 10, 24, 25]. Exosomes carry various cargo molecules, such as functional proteins, miRNAs, and signal lipids [26, 27], which can mediate cell-to-cell communication by initiating a series of biological responses in recipient cells. After these exosomes are secreted, the recipient cells take up these exosomes through phagocytosis/endocytosis or fusion, thereby receiving cargo.

In this study, we evaluated the effects of UCB-MSCs and UCB-MSC-derived exosomes (UCB-MSC-exo) on scar formation during wound healing in rats. We revealed that the therapeutic capacity of UCB-MSCs on wound healing could be achieved by exosomes. Additionally, we identified two specific miRNAs carried by UCB-MSC-exo as essential factors that inhibit fibroblast differentiation into myofibroblasts via inhibition of TGF-β receptor activity. The results suggest that UCB-MSC-exo might represent a novel strategy to prevent scar formation and improve skin appendage regeneration during wound healing in the clinic.

## Materials and methods

### Cell culture and characterization

Human UCB-MSCs were a gift from the Bethune First Hospital of Jilin University and were used with informed consent. The Medical Ethics Committee of Hospital of Stomatology of Jilin University approved the research about UCB-MSCs (2020[42]). The protocol for UCB-MSC isolation was processed as previously described [18]; briefly, umbilical cord blood was collected from delivered placentas using 50 ml syringes (contained 1000 U of heparin) and then diluted with PBS. Mononuclear cells were isolated by density gradient centrifugation using Ficoll-Hypaque-Plus solution (Solarbio, China). Freshly isolated mononuclear cells were suspended in Dulbecco’s modified Eagle’s medium (Hyclone, USA) containing 10% fetal bovine serum (Hyclone, USA) and 1% penicillin/streptomycin (Biological Industries, Israel) and incubated at 37 °C in 5% CO_2_. We continued the cultures for another 7 days after fibroblast-like cells appeared on the bottom of the flasks. Then, these fibroblast-like cells were collected and expanded. The second to fifth passage cells were used for experiments.

UCB-MSCs were characterized by surface marker profiling (CD73, CD90, and CD105) via flow cytometry (FCM) and immunofluorescence (IF) staining, as previously described [28]. For FCM, the cell suspension was stained with primary antibodies, washed three times with PBS, stained with secondary antibodies, washed three times with PBS, and quantitatively analyzed using a BD FACSCelesta (BD Biosciences, USA). Table S1 lists the antibodies used. The process of IF staining is described in the following section. The multipotency of UCB-MSCs was detected via inducing adipogenic (Oil Red-O staining), osteogenic (Alizarin Red S staining), and chondrogenic (Alizarin Blue staining) differentiation.

The human dermal fibroblasts (HDFs) and epidermal stem cells (EPSCs) were purchased commercially (Dingguo, Beijing, China). The cell lines used in this study were all cultured in DMEM containing 10% fetal bovine serum and 1% penicillin/streptomycin and incubated at 37 °C in 5% CO_2_.

### Exosome preparation

**Fig. 1:**
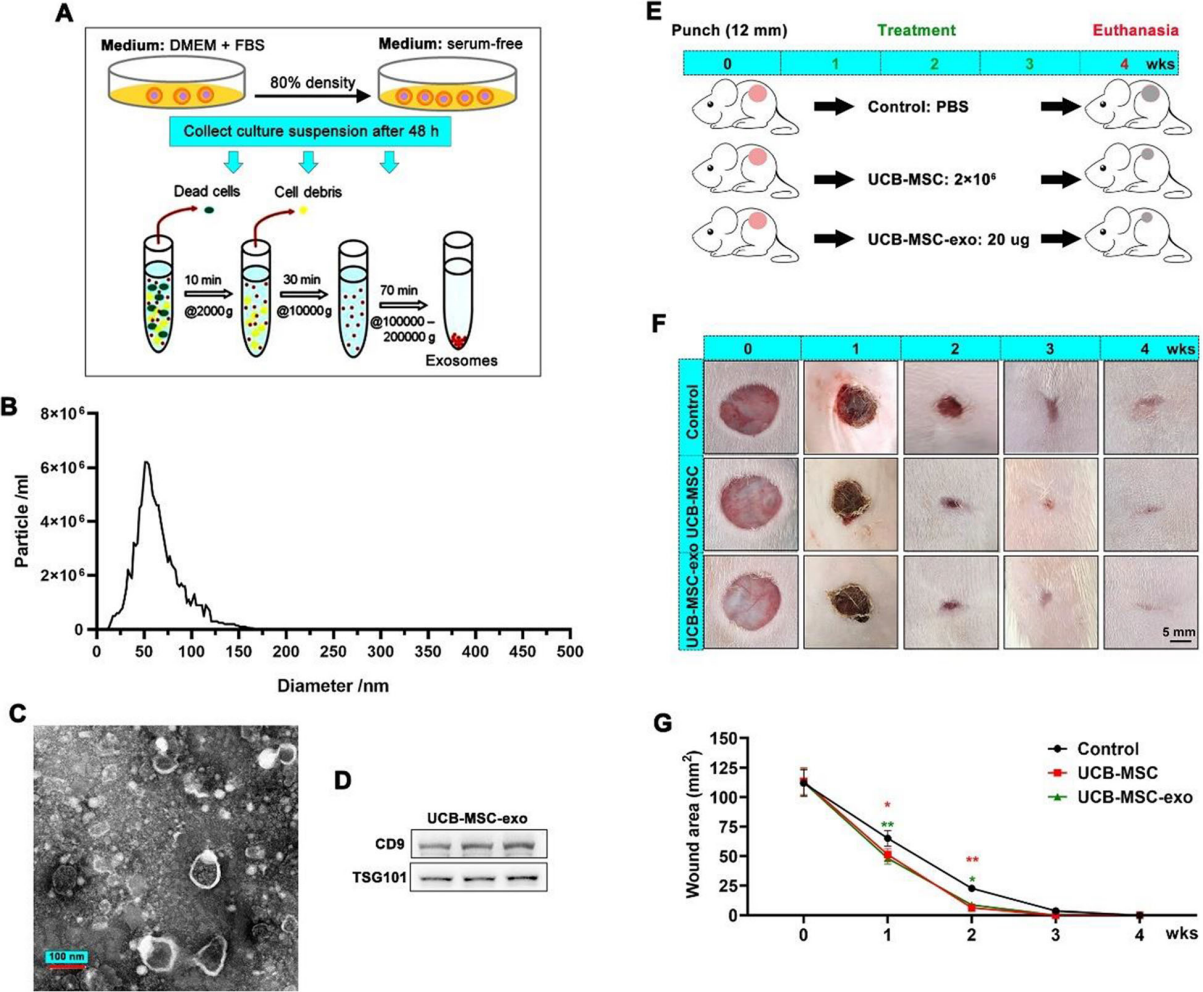
UCB-MSC-exo accelerate wound closure and suppress scar formation in full-thickness skin-wounded rats. **A** Schematic representation of UCB-MSC-exo preparation. **B** UCB-MSC-exo particle size was measured using NanoSight. **C** Morphological characteristics of UCB-MSC-exo were measured using transmission electron microscopy (scale bar = 100 nm). **D** Exosomal markers of UCB-MSC-exo were measured via western blot. **E** Schematic representation of animal experiments. The full-thickness excisional wound (12 mm diameter) was created on the dorsal region. The rats were then administered by tail vein injection every week: PBS (control group), UCM-MSCs (2 × 10^6^ cells), or UCB-MSC-exo (20 μg). **F** Morphological changes during wound healing; scale bar = 5 mm. **G** Changes in the wound area during wound healing. ^*^p < 0.05; ^**^p < 0.01; means ± SD; *n* = 5

UCB-MSCs (passage 4 to passage 8) were seeded into T175-cell culture flasks and allowed to reach 70% to 80% confluence. The media was then replaced by a serum-free medium (Hyclone, USA), and the cells were cultured for another 48 h. Next, the conditioned medium was collected and centrifuged to remove the dead cells and debris, and then, collected exosomes were washed three times and stored at − 80 °C (Fig. 1A). UCB-MSCs were characterized using a NanoSight NS300 (Malvern Instruments, UK), transmission electron microscopy, and Western blot of CD9 and TSG101 [9].

### In vivo wound generation and UCB-MSC-exo treatment

Female SD rats (6–8 weeks old; 220–250 g) were purchased from Liaoning Changsheng Biotechnology Co., Ltd. (Benxi, China). The animal experiment protocol and procedure were approved by the Animal Experiment Ethics Committee of Jilin University (approval no.: SY202008010). Rats were randomly assigned to UCB-MSC, UCB-MSC-exo, or control groups (10 rats/group). All rats were anesthetized by 3% pentobarbital sodium (30 mg/kg) before surgery. A 12-mm diameter excisional wound was created on the dorsal region under sterile surgical conditions. The rats were treated with UCB-MSCs (2 × 10^6^ cells) or UCB-MSC-exo (20 μg) by tail vein injection (100 μl). Rats in the control group were injected with an equivalent volume of PBS. The above treatment was conducted weekly. The extent of wound healing was photographed every week. Rats were sacrificed 2 or 4 weeks after surgery, and the healing skin tissue was collected for histological and quantitative real-time polymerase chain reaction (qRT-PCR) analysis.

### Cell counting kit-8 (CCK8) assay

EPSCs or HDFs were seeded into 96-well plates at a density of 10,000 cells/well (100 μl) and cultured for 24 h, and then UCB-MSC-exo (25 ng/ml) were added to the culture for another 24 h. To each well was added 10 μl CCK-8 solution (MedChemExpress, Shanghai, China), and then cultures were incubated for 4 h in an incubator. Absorbance was measured at 450 nm using a microplate reader.

### Cell scratch assay

EPSCs or HDFs were seeded into 24-well plates at a density of 50,000 cells/well (500 μl) and cultured for 24 h. The cell monolayer was scraped along a straight line with a P-200 pipette tip to form a “scratch”. The debris was removed, the edge of the scratch was smoothed by washing the cells with PBS, and then the medium was replaced with 200 μl of medium containing 25 ng/ml UCB-MSC-exo.

### In vitro myofibroblast differentiation induction and UCB-MSC-exo/miRNA-mimic treatment

HDFs were seeded into 24-well plates at a density of 50,000 cells/well (500 μl) combined with TGF-β1 (25 ng/ml) and cultured for 48 h to induce myofibroblast differentiation. At the same time, UCB-MSC-exo (25 ng/ml) or miRNA mimics (50 nM) were added to each well. The expression of Collagen I, α-SMA, *TGFBR1*, and *TGFBR2* was measured by IF staining and qRT-PCR analysis.

### Histopathological analysis

Healing skin was fixed with 4% paraformaldehyde solution, dehydrated by graded ethanol, and embedded in paraffin. The sections were cut into slices and stained with hematoxylin and eosin (H&E) or Masson and photographed under a microscope (Precipoint M8, Germany). The numbers of skin appendages and the levels of collagen fibers (blue) and myofibers (red) in the wounded area were calculated with Image-Pro Plus software. For IF staining, rehydrated antigen-repaired paraffin sections or fixed cells were incubated with primary antibodies and conjugated secondary antibodies, stained with 4′,6-diamidino-2-phenylindole dihydrochloride (DAPI) (Beyotime, China) and photographed under a microscope (VOS M5000, USA). Table S1 lists the antibodies used.

### qRT-PCR

Total RNA was isolated from healing skin, HDFs, and UCB-MSC-exo using TRIzol reagent (Sangon Biotech Co., Ltd., Shanghai, China). cDNA was synthesized from total RNA using the cDNA Synthesis Kit (Takara, Japan). qRT-PCR analysis was performed using SYBR Green Master (Roche, Switzerland) in an ABI 9700 Detection System (Thermo Fisher Scientific, MA, USA). *GAPDH* mRNA was used as an internal control. qRT-PCR for miRNA was performed using the Bulge-Loop^TM^ miRNA qRT-PCR Starter Kit (Ribobio, China) according to the manufacturer’s instructions. *U6* small RNA was used as an internal control. Table S2 lists the primers used. All experiments were repeated in triplicate.

### Statistical analysis

All quantitative data are shown as means ± SD (*n* ≥ 3). Statistical analysis was conducted using Graphpad Prism software, and significant differences were evaluated using a one-way analysis of variance; *p* < 0.05 was considered statistically significant.

## Results

### UCB-MSC-exo accelerate wound closure and suppresses scar formation in full-thickness skin-wounded rats

UCB-MSCs expressed the putative mesenchymal markers CD73, CD90, and CD105 and could be induced to adipogenic, osteogenic, and chondrogenic differentiation (Figure S1). These results confirmed the phenotypic characterization of cells as MSCs. UCB-MSC-exo were prepared from UCB-MSC-conditioned medium by gradient centrifugation (Fig. 1A). It was characterized by a discoid morphology (Fig. 1B) with a diameter of 30–150 nm (Fig. 1C) determined by transmission electron microscopy and NanoSight. The result of Western blot showed that UCB-MSC-exo expressed exosomal markers CD9 and TSG101 (Fig. 1D).

The therapeutic potentials of UCB-MSCs and UCB-MSC-exo were compared in full-thickness skin-wounded rats (Fig. 1E). As expected, there were no significant differences in wound closure and scar area between the UCB-MSC-exo and UCB-MSC groups; however, UCB-MSC-exo and UCB-MSC significantly accelerated wound closure and smoothed wound edges, resulting in a smaller scar size than in the control group (Fig. 1F, G). These results suggest that UCB-MSCs accelerate wound closure and suppress scar formation, which may function via UCB-MSC-exo.

### UCB-MSC-exo improve regeneration and regulates collagen distribution in wound healing of rats

**Fig. 2:**
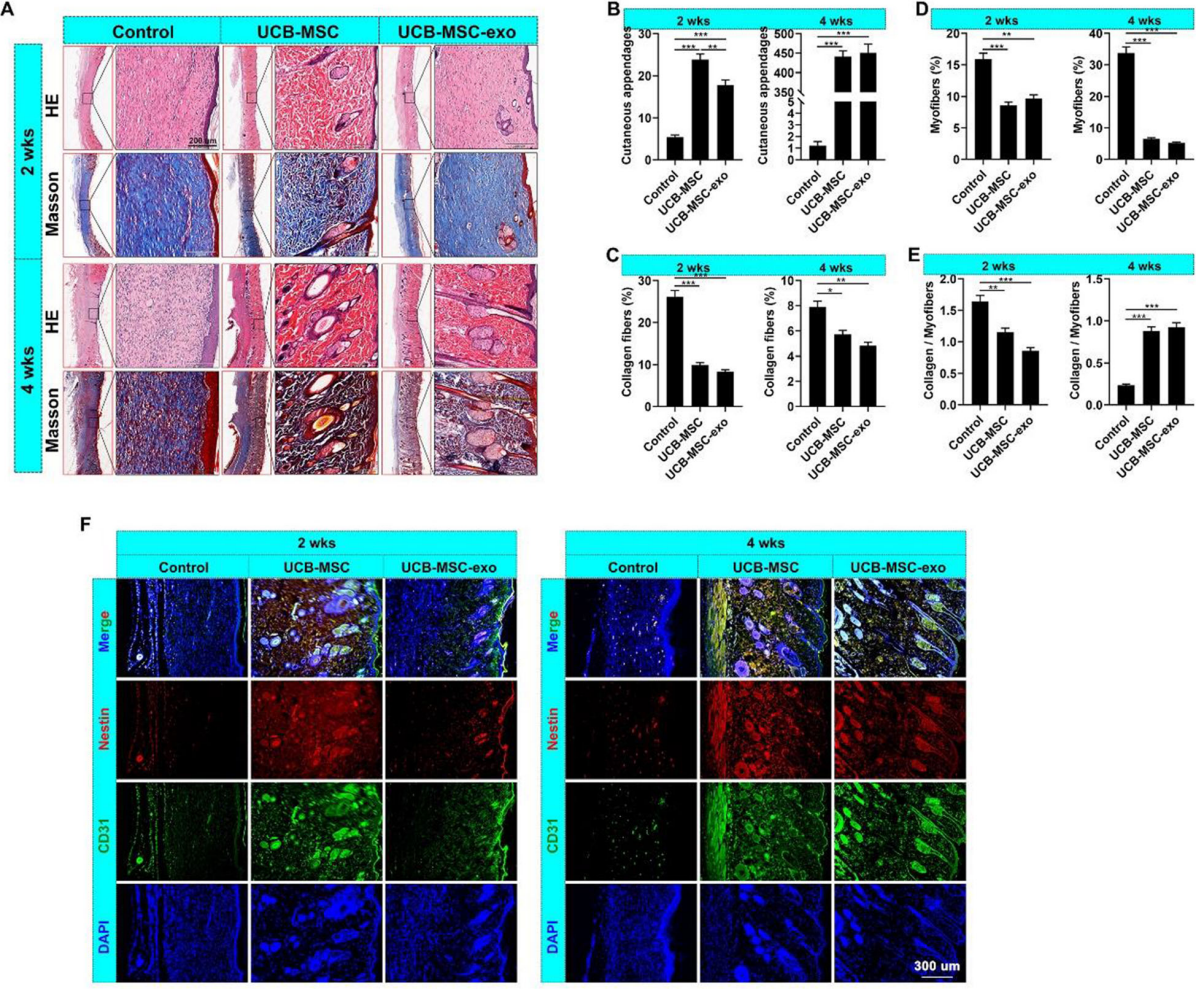
UCB-MSC-exo improve the regenerative wound healing in rats. **A** H&E and Masson staining of the healing skin; scale bar = 200 μm. **B** Number of skin appendages (hair follicles and sebaceous glands) in the healing skin. **C**–**E** Levels of collagen fibers (blue), myofibers (red) and the ratio of collagen fibers to myofibers in the healing skin, per Masson staining. **F** IF staining of Nestin (nerves) and CD31 (vessels) in the healing skin; scale bar = 300 μm. ^*^p < 0.05; ^**^p < 0.01; ^***^p < 0.001; means ± SD; *n* = 3

Pathological analysis via H&E staining showed that healing skin in the UCB-MSC and the UCB-MSC-exo groups exhibited more appendages than that in the control group. Interestingly, the skin in the UCB-MSC group exhibited significantly (*p* < 0.01) more appendages than that in the UCB-MSC-exo group after 2 weeks of treatment; however, there were no significant differences after 4 weeks of treatment (Fig. 2A–C). There were also significantly (*p* < 0.05) fewer collagen fibers (blue) and myofibers (red) in both the UCB-MSC and the UCB-MSC-exo groups than in the control group by Masson staining (Fig. 2A, C, D). Moreover, the ratios of collagen fibers to myofibers among the groups displayed different trends after 2 and 4 weeks of treatment (Fig. 2E); the ratios in the UCB-MSC and UCB-MSC-exo groups were lower than those of the control group after 2 weeks of treatment and higher after 4 weeks of treatment.

Furthermore, we evaluated the effects of UCB-MSC-exo on nerve and vessel regeneration in healing skin. IF staining results showed that, after 2 or 4 weeks of treatment, the skin in the UCB-MSC-exo and UCB-MSC groups exhibited significantly more vessels (CD31^+^) and nerves (nestin^+^) than that in the control group (Fig. 2F). These results suggest that UCB-MSCs and UCB-MSC-exo have similar effects on skin regeneration, promoting the regeneration of skin appendages, vessels, and nerves, decreasing fiber formation, and regulating the ratio of collagen fiber to myofiber in the wound healing process.

### UCB-MSC-exo promote the proliferation and migration of skin cells in vivo and in vitro

**Fig. 3:**
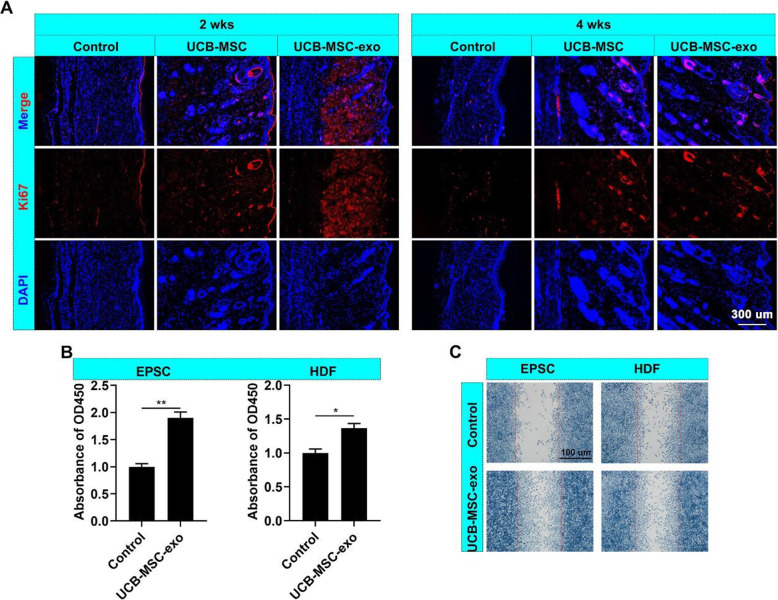
UCB-MSC-exo promote the proliferation and migration of skin cells. **A** IF staining of Ki67 in the healing skin; scale bar = 300 μm. **B**, **C** Effects of UCB-MSC-exo (25 ng/ml) on proliferation and migration of EPSCs and HDFs using the CCK8 and cell scratch assays in vitro; scale bar = 100 μm; ^*^p < 0.05; ^**^p < 0.01; means ± SD; *n* = 3

To investigate the mechanism by which UCB-MSC-exo accelerate wound closure, we examined the effects of UCB-MSC-exo on the proliferation and migration of skin cells in vivo and in vitro (Fig. 3). The results showed that the cell division in healing skin in the UCB-MSC-exo group was higher than that in the control group (Fig. 3A). Additionally, UCB-MSC-exo also promoted the proliferation and migration of EPSCs and HDFs in vitro (Fig. 3B–C). These results suggest that UCB-MSC-exo accelerate wound closure, which can occur, in part, via the promotion of the proliferation and migration of skin cells during wound healing.

### UCB-MSC-exo suppress scar formation by inhibiting myofibroblast differentiation

**Fig. 4:**
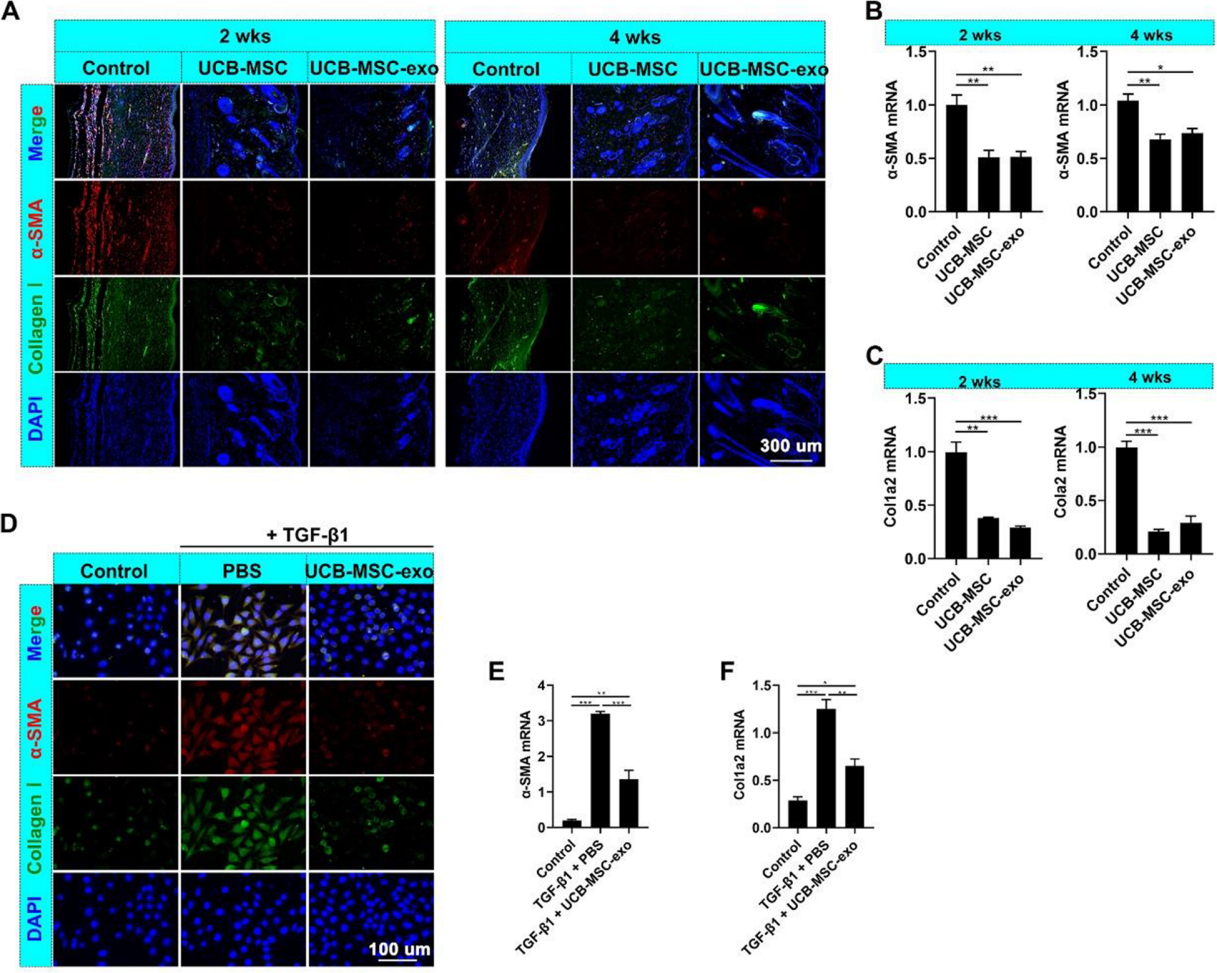
UCB-MSC-exo suppress myofibroblast formation. **A** IF staining of α-SMA and collagen I in the wound healing skin; scale bar = 300 μm. **B**, **C** mRNA levels of *α-SMA* and *Col1a2* in the wound healing skin. HDFs were cultured for 48 h with TGF-β1 (25 ng/ml) to induce myofibroblast differentiation. Some cells were also treated with UCB-MSC-exo (25 ng/ml). **D**–**F** IF staining and qRT-PCR analysis of α-SMA and collagen I in HDFs; scale bar = 100 μm. ^*^p < 0.05; ^**^p < 0.01; ^***^p < 0.001; means ± SD; *n* = 3

α-SMA and collagen I are critical markers of myofibroblasts and scar tissue. The results showed that α-SMA and collagen I were highly expressed in the healing skin; however, the expression levels were reduced by UCB-MSC-exo treatment (Fig. 4A–C). To further verify these results, HDFs were cultured in the presence of TGF-β1 to induce myofibroblast differentiation in vitro, and UCB-MSC-exo (25 ng/ml) were used to intervene. Expectedly, UCB-MSC-exo treatment strongly inhibited the TGF-β1-induced high expression of α-SMA and collagen I (Fig. 4D–F). These results suggest that UCB-MSC-exo may suppress scar formation by inhibiting myofibroblast differentiation.

### UCB-MSC-exo-derived miRNAs inhibit myofibroblast differentiation via targeting TGF-β receptor

**Fig. 5:**
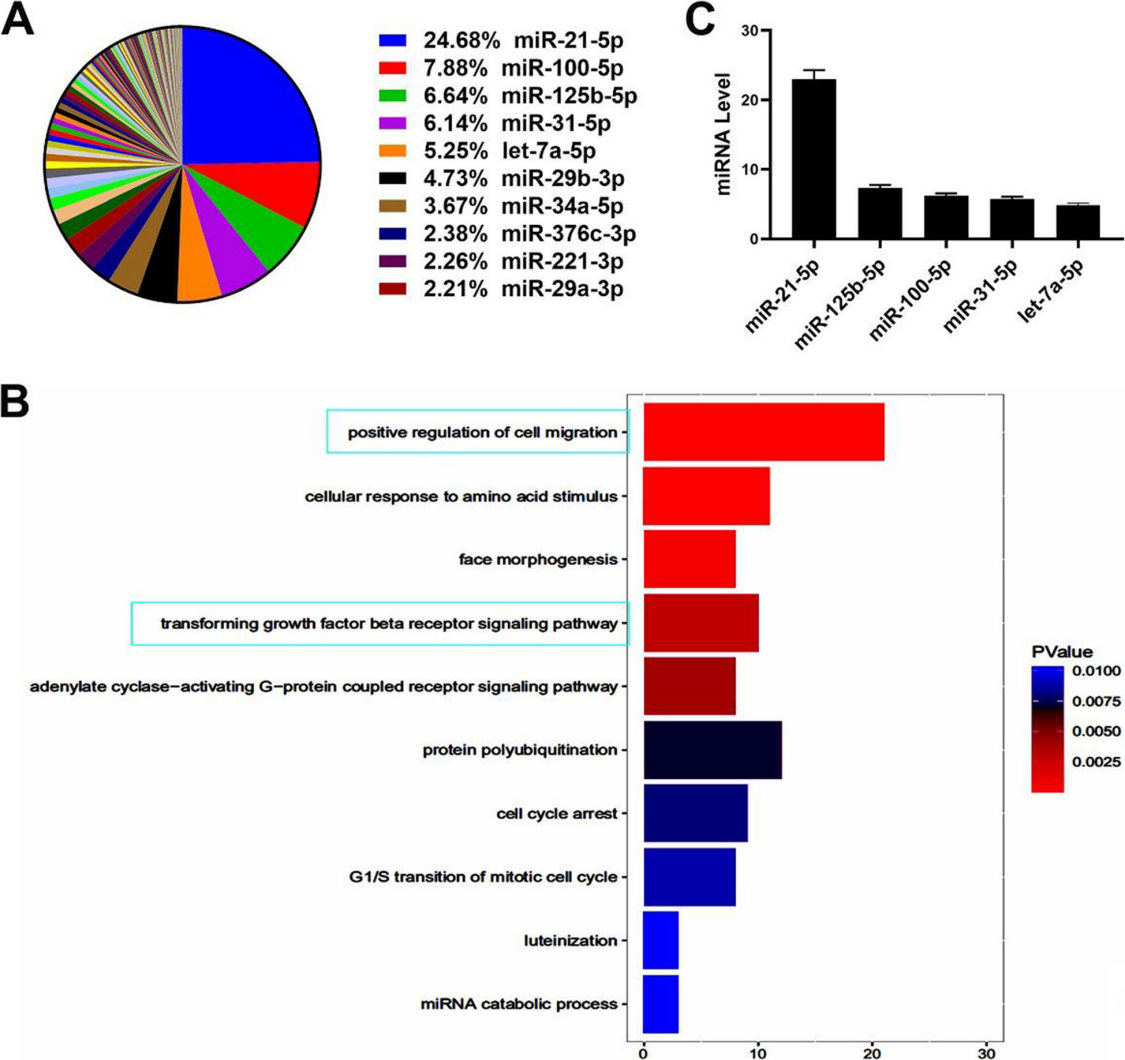
Functional analysis of UCB-MSC-derived miRNAs and identification of UCB-MSC-exo-derived miRNAs. **A** UCB-MSC-miRNA abundance analysis according to Meng X et al. The top 10 abundant miRNAs in UCB-MSC are color-labeled. **B** Target mRNA prediction of UCB-MSC-miRNAs via Gene Ontology analysis; the TGF-β signaling pathway was highly related to myofibroblast formation. **C** UCB-MSC-exo-miRNAs, enriched in UCB-MSCs, measured by qRT-PCR analysis

**Fig. 6:**
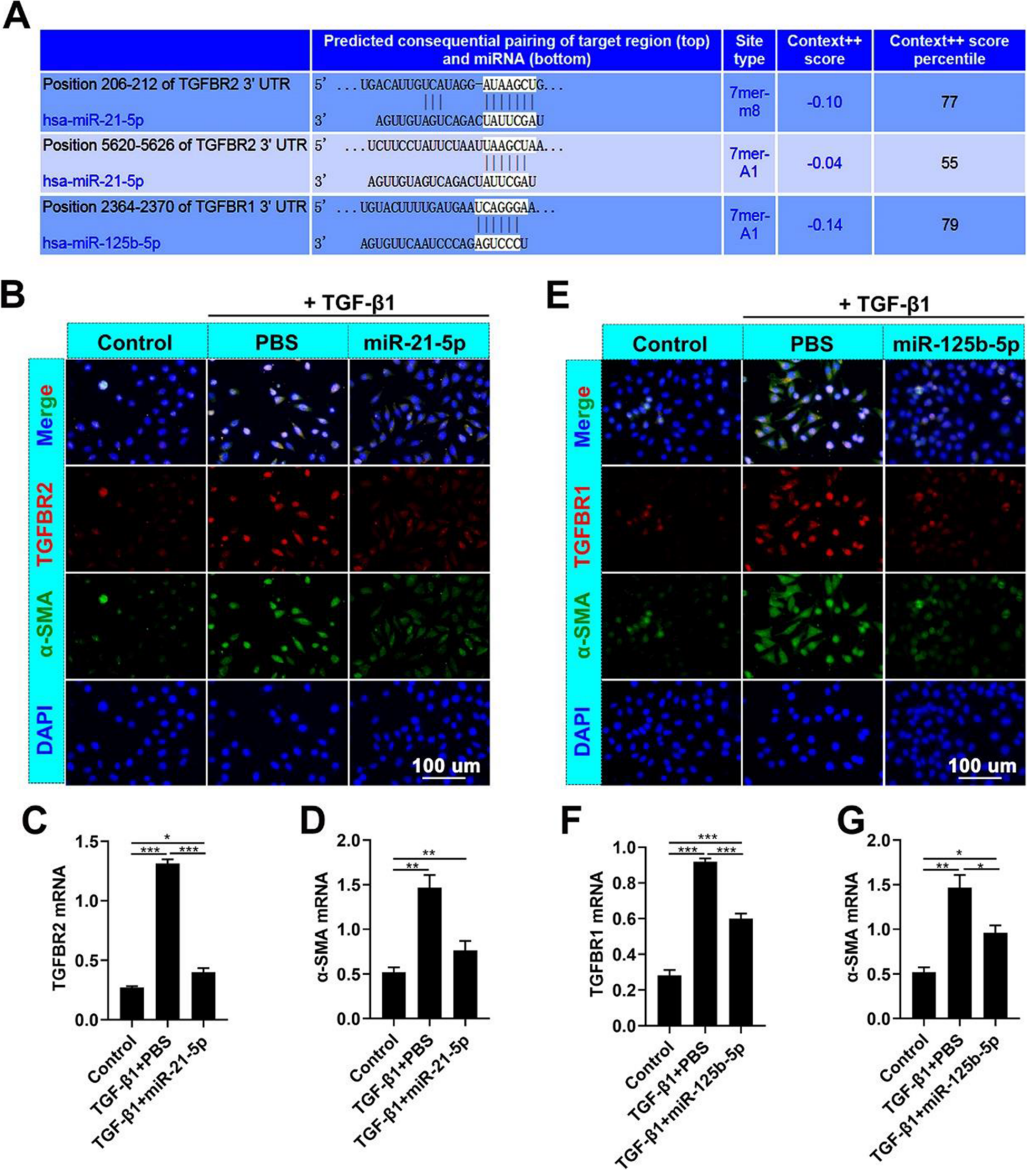
UCB-MSC-exo-derived specific miRNAs target TGF-β receptors to inhibit myofibroblast differentiation. **A** List of predicted binding sites for UCB-MSC-exo-miRNAs and their targets. **B**–**D** Expression levels of *TGFBR2* and *α-SMA* in HDFs treated with miR-21-5p-mimics. **E**–**G** Expression levels of TGF-βR1 and α-SMA in HDFs treated with miR-125b-5p-mimics; scale bar = 100 μm; ^*^p < 0.05; ^**^p < 0.01; ^***^p < 0.001; means ± SD; *n* = 3

miRNAs are the main components of exosomal functional RNA [9, 10]. We analyzed the miRNAs in UCB-MSCs as reported by Meng et al. [28] (Fig. 5A). To further reveal the possible roles of these miRNAs, we used Gene Ontology analysis to predict their function. We demonstrated that these miRNAs were positively correlated with the TGF-β signaling pathway (Fig. 5B). Generally, MSC- and their exosome-derived miRNAs were highly correlated; thus, we detected the levels of miRNAs in UCB-MSC-exo, which were similarly highly expressed in UCB-MSCs. We found that miR-21-5p, miR-125b-5p, miR-100-5p, miR-31-5p, and let-7a-5p were highly expressed in UCB-MSC-exo (Fig. 5C). Moreover, we predicted the target genes of these miRNAs using TargetScan (http://www.targetscan.org/) and found that miR-21-5p and miR-125b-5p were predicted to target *TGFBR2* and *TGFBR1* mRNAs directly, respectively (Fig. 6A).

**Fig. 7:**
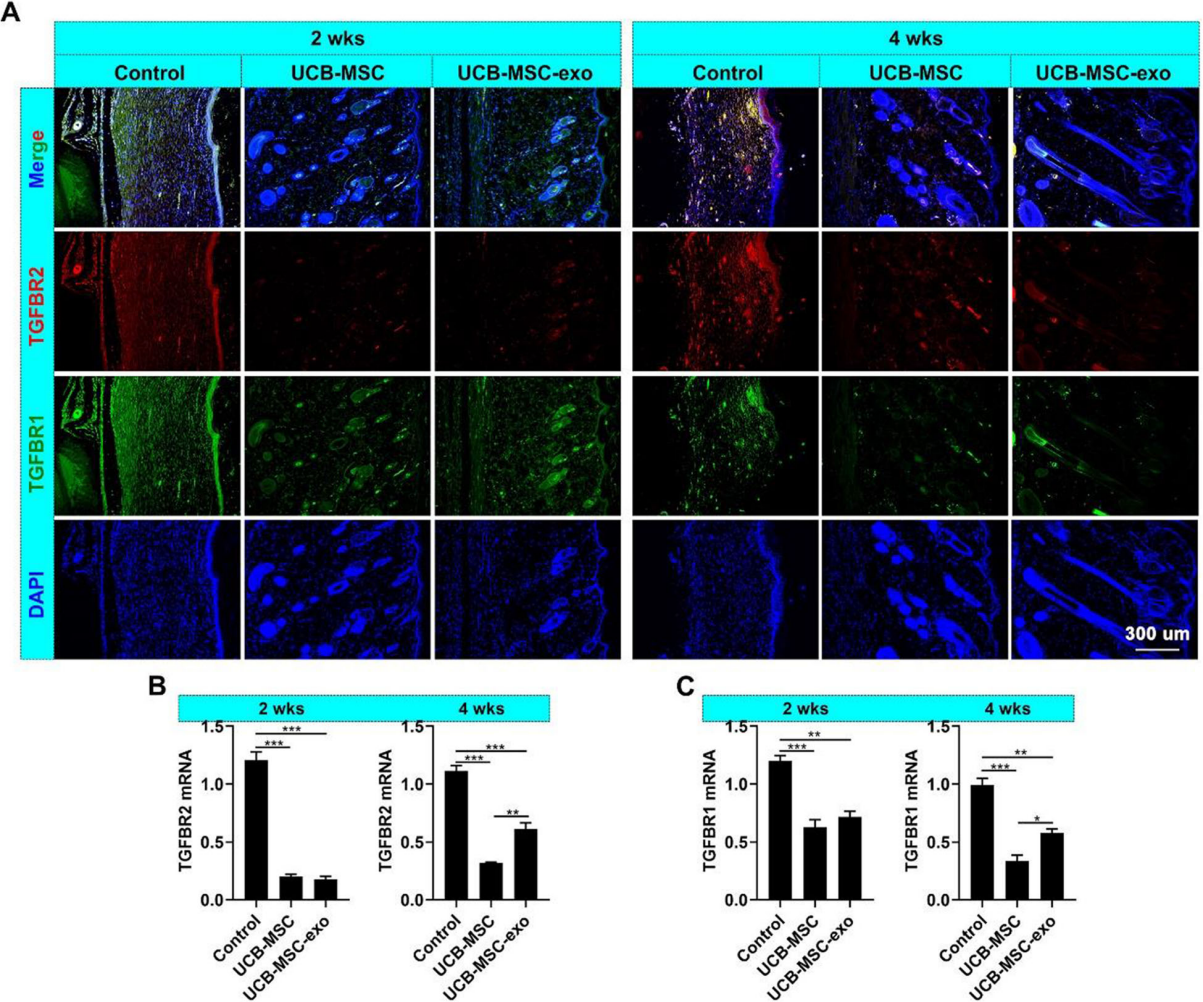
UCB-MSC-exo inhibit *TGFBR2* and *TGFBR1* expression in wound healing in rats. **A** IF staining of TGFBR2 and TGFBR1 in the wound healing skin. **B**, **C** mRNA levels of *TGFBR2* and *TGFBR1* in the healing skin; scale bar = 300 μm; ^*^p < 0.05; ^**^p < 0.01; ^***^p < 0.001; means ± SD; *n* = 3

miRNA mimics were added into the HDF (+TGF-β1) culture system to determine whether or not these miRNAs could affect the expression of TGFBR2 and TGFBR1 and thus verify the roles of miR-21-5p and miR-125b-5p in UCB-MSC-exo function. The results showed that miR-21-5p and miR-125b-5p significantly inhibited *TGFBR2* and *TGFBR1*, respectively, and decreased the expression of α-SMA (Fig. 6B–G). Moreover, the expression levels of *TGFBR2* and *TGFBR1* in the healing skin of rats were also reduced by UCB-MSC-exo treatment (Fig. 7). These results suggest that UCB-MSC-exo suppress myofibroblast differentiation, which can be, in part, via the expression of specific miRNAs miR-21-5p, targeting *TGFBR2*, and miR-125b-5p, targeting *TGFBR1*.

## Discussion

Adult wound healing generally comprises scar repair, which may be the result of excessive myofibroblast formation [1, 2]. MSCs are a promising technique to accelerate wound closure and limit scarring in wound healing [9, 14–16]. UCB-MSCs have been used to accelerate the closure of diabetic wounds and have been reported to promote the expression of anti-scarring factors [17–21]. However, few attempts have been made to study the effects of UCB-MSCs on scar formation. More and more studies have shown that MSCs play a role mainly through immune regulation and paracrine processes, rather than directly differentiating [9, 10, 14, 29]. UCB-MSC-exo are the primary source of UCB-MSC paracrine factors; it remains unclear whether these are the main effectors of UCB-MSC function, especially as the roles of UCB-MSC-exo-derived “cargo” is unknown. For the first time, the present study has demonstrated that UCB-MSCs accelerate wound closure and suppress scar formation in wound healing, which can be, in part, via UCB-MSC-exo. We also identified two specific miRNAs carried by UCB-MSC-exo that could inhibit the signaling activities of TGF-β receptors, thereby inhibiting myofibroblast differentiation. To a certain extent, our research proposes a new strategy for the clinical application of UCB-MSCs to prevent scar formation.

In wound healing, MSC-based therapy is a promising strategy to enhance re-epithelialization, inhibit fibrotic remodeling, promote regeneration of skin appendages, increase angiogenesis, stimulate endogenous stem cell recruitment, and modulate inflammation [9, 10, 14–16, 29]. Additionally, UCB-MSCs might represent a more promising option that has the potential to overcome several limitations, including greater expansion capacity with differentiation potential, lower immunogenicity compared with adult-derived MSCs, and easier obtainment compared to ESCs, due to related ethical issues [22, 23].

UCB-MSCs have been used to enhance re-epithelialization and improve the quality of wound healing. Jae-A Jung et al. [19] and N Çil et al. [20] respectively reported that UCB-MSCs promote diabetic wound healing by increasing collagen synthesis and neovascularization. Moreover, Hanako Doi et al. [18] found that UCB-MSCs express lower levels of IL-1 pro-inflammatory cytokines and higher levels of MMP1 and PLAU ECM degradation enzymes than Wharton’s jelly MSCs, suggesting that UCB-MSCs were more likely to favor scarless wound healing. However, they failed to significantly reduce scar formation following direct injection of UCB-MSCs into full-thickness skin-wounded nude mice [18]. In the present study, we evaluated the essential roles of UCB-MSCs in the wound healing of rats. We observed the phenomena of earlier wound closure, smaller scar formation, and more skin appendages, nerves, and vessels in healing skin in the UCB-MSC group compared with those in the control group. Myofibroblast differentiation and scar formation were also inhibited by UCB-MSC treatment, as indicated by the decreased expressions of α-SMA and collagen I in the healing skin of rats. Classically, α-SMA is considered a marker of myofibroblast differentiation [30, 31], and collagen I is considered the main component of scar tissue [2, 3]. The over-differentiation of myofibroblasts and the continuous expression of collagen I is a fundamental cause of scar formation [2, 3]; thus, intervention is required during wound healing to prevent myofibroblast accumulation rather than taking remedial measures after scar formation. This study observed different in vivo experimental results from those observed by Hanako Doi et al. [18]. This might be due to differences in UCB-MSC treatment methods (tail vein injection versus local injection around the wound), suggesting that the diseased microenvironment is not conducive to MSC survival and retention, limiting treatment efficacy. We also observed that UCB-MSCs could inhibit collagen deposition, which is counter to the results of Jae-A Jung et al. [19], perhaps due to the different animal wound models used (mechanical wounds versus diabetic wounds), as diabetic wound healing is different from mechanical wound healing, it may be in the early stage of healing for a long time. It is known that MSCs can regulate collagen remodeling to inhibit scar hyperplasia. In an early stage, MSCs promote collagen remodeling through the synthesis of types I and III, whereas they reduce scarring in the late stage by inhibiting collagen formation [32]. This may suggest that UCB-MSCs have a flexible targeting effect in different pathologies, which may increase the scope of its application.

It is currently believed that MSCs play a therapeutic role in vivo, mainly through paracrine signaling [9, 10, 14, 29]. MSCs can release biologically active molecules to affect the proliferation, migration, and survival of receptor cells. Our recent studies have reported that EPSCs [9] and amniotic fluid stem cells [10] promote skin regeneration via secreted exosomes. In this study, we also evaluated the essential roles of UCB-MSC-exo in the wound healing of rats and found that the effect of UCB-MSC-exo was similar to that of UCB-MSCs, suggesting that the regenerative healing effect of UCB-MSC on wound healing may be achieved via exosome secretion. We tracked UCB-MSC in vivo and detected whether they exist on the wounded site 1 week and 2 weeks after transplantation. Interestingly, we found that UCB-MSC aggregation was detected at the wounded site 1 week after cell implantation. However, there are indeed very few UCB-MSCs after cell implantation after 2 weeks (data not shown), suggesting that few UCB-MSCs become permanently engrafted within the repaired tissue. Besides the regenerative repair of skin wounds to achieve scar-free healing, rapid wound closure is also critical because wound closure is essential to block external environmental interference. Although the contractility of myofibroblasts is beneficial to wound closure in the early stage, the inhibition of UCB-MSC-exo on myofibroblast differentiation does not necessarily lead to a slowdown in wound healing. We found that UCB-MSCs can promote the proliferation and migration of skin cells, including EPSCs and HDFs, which may be essential for accelerating wound closure; this is similar to the results reported by Yoon-jin Kim et al. [19]. UCB-MSCs and UCB-MSC-exo have similar repair effects on wound healing; however, we believe that UCB-MSC-exo may have other advantages, such as overcoming the immune and tumorigenic issues caused by allotransplantation.

Exosomes generally play a crucial role in cell communication, transmitting information to neighboring cells through their “cargo,” including proteins, DNA, mRNAs and miRNAs [26, 27, 33]. The therapeutic potential of exosomes depends on the composition of the “cargo” they carry [33]. It has been reported that miRNAs are generally abundant in exosomes [9, 10]; these miRNAs regulate various signaling pathways in the receptor cells, including cell trafficking, apoptosis, angiogenesis, and proteolysis via targeting transcription factors and genes [33]. We analyzed the miRNAs in UCB-MSCs as reported by Meng et al. [28] and found that these miRNAs were positively correlated with the TGF-β signaling pathway. Generally, the MSC-derived miRNAs and their exosome-derived miRNAs are highly correlated, which is confirmed by our results. We found that the effect of anti-myofibroblast differentiation may result from a pair of TGF-β receptor-targeting miRNAs, miR-21-5p, and miR-125b-5p. miR-125b-5p has been reported to suppress liver fibrosis in non-alcoholic fatty liver disease by inhibiting the RhoA signaling pathway [34]. In our study, miR-125b-5p also suppressed fibrosis scarring by inhibiting *TGFBR1* expression. Additionally, miR-21-5p could inhibit *TGFBR2* expression in the TGF-β signaling pathway, thereby suppressing myofibroblast differentiation, in contrast to other reports, in which miR-21-5p promoted gastric cancer and kidney fibrosis by upregulating Smad7 in the TGF-β signaling pathway [35, 36]. Generally, miRNAs can target different mRNAs simultaneously. Therefore, we believe that miR-21-5p may be a double-edged sword regulating TGF-β signaling; its function may vary according to the cell state and the specific molecular network involved. Based on the above results, we suggest that UCB-MSC-exo miRNAs may be important regulators of the TGF-β signaling pathway by inhibiting myofibroblast differentiation during wound healing in the skin.

## Conclusions

**Fig. 8:**
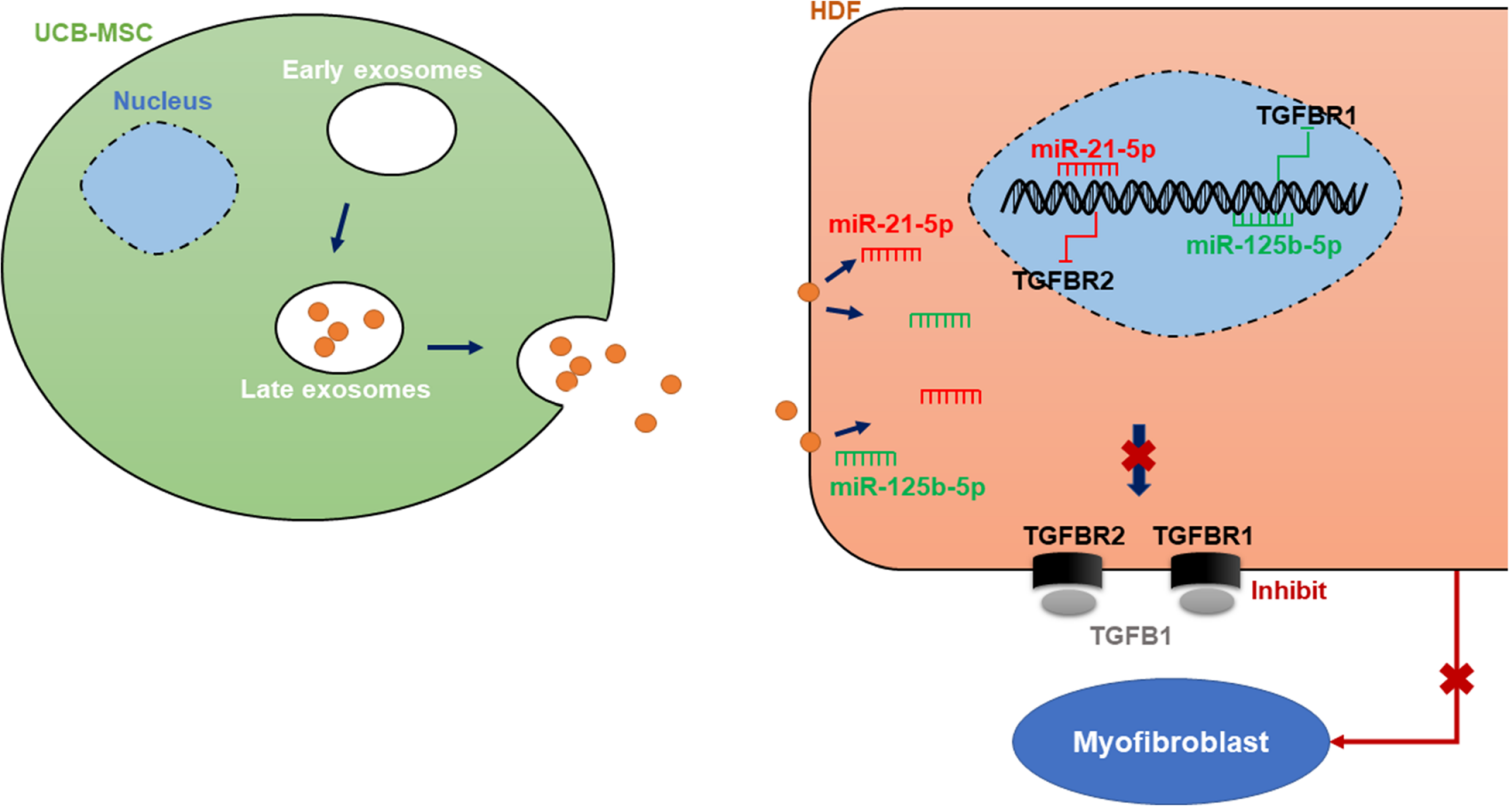
UCB-MSC-exo improve regenerative wound healing and suppress scar formation by inhibiting the expression of TGF-β receptors. When UCB-MSCs are transplanted into the skin wound, multiple vesicles bud from the plasma membrane, releasing the exosomes into the extracellular space where they then fuse with the plasma membrane of receptor cells. UCB-MSC-exo, carrying cargos of proteins, and RNAs, can travel to and be internalized by recipient cells (HDFs). Then, UCB-MSC-exo-derived specific miRNAs (such as miR-21-5p and miR-125b-5p) can regulate gene expression in HDFs. miR-21-5p targets *TGFBR2*, whereas miR-125b-5p targets *TGFBR1*, thereby suppressing myofibroblast differentiation, and ultimately reducing scar formation and promoting regenerative wound healing

The present study revealed that UCB-MSCs could stimulate regenerative wound healing via their exosomes. Through exosome-mediated intercellular transfer, miR-21-5p and miR-125b-5p derived from UCB-MSC-exo inhibited *TGFBR2* and *TGFBR1*, respectively, thereby inhibiting the TGF-β signaling pathway to suppress myofibroblast differentiation (Fig. 8). As an alternative to cell therapy, UCB-MSC-exo might represent a novel strategy to prevent scar formation during wound healing in the clinic.

## Supplementary Information

**Additional file 1: Figure S1.** Phenotypical characterization of UCB-MSCs. **A** FCM analysis of expression profiles of cell surface markers CD73, CD90, and CD105. **B** IF staining analysis expression profiles of cell surface markers CD73, CD90, and CD105; scale bar = 300 μm. **Table S1.** Antibodis. **Table S2.** Primers.

**Additional file 2.** Gene ontology.

## Data Availability

The datasets used and/or analyzed during the present study are available from the corresponding author on reasonable request.

## Abbreviations

α-SMA: α-Smooth muscle actin
CCK8: Cell counting kit
EPSC: Epidermal stem cells
FCM: Flow cytometry
H&E: Hematoxylin and eosin
HDF: Human dermal fibroblasts
IF: Immunofluorescence staining
PBS: Phosphate-buffered saline
TGF-β1: Transforming growth factor-β1
TGFBR1: TGF-β receptor type I
TGFBR2: TGF-β receptor type II
qRT-PCR: Quantitative real-time polymerase chain reaction
UCB-MSC: Umbilical cord blood mesenchymal stem cell
UCB-MSC-exo: UCB-MSC-derived exosomes
