# Supplementary material for: Exosomes derived from human umbilical cord blood mesenchymal stem cells stimulate regenerative wound healing via transforming growth factor-β receptor inhibition
Source: Stem Cell Res Ther. 2021 Aug 3;12:434. doi: 10.1186/s13287-021-02517-0 (PMC8336384; doi:10.1186/s13287-021-02517-0)
Supplement: Supplementary file 1 — Additional file 1: Figure S1. Phenotypical characterization of UCB-MSCs. A FCM analysis of expression profiles of cell surface markers CD73, CD90, and CD105. B IF staining analysis expression profiles of cell surface markers CD73, CD90, and CD105; scale bar = 300 μm. Table S1. Antibodis. Table S2. Primers. [file 13287_2021_2517_MOESM1_ESM.docx]

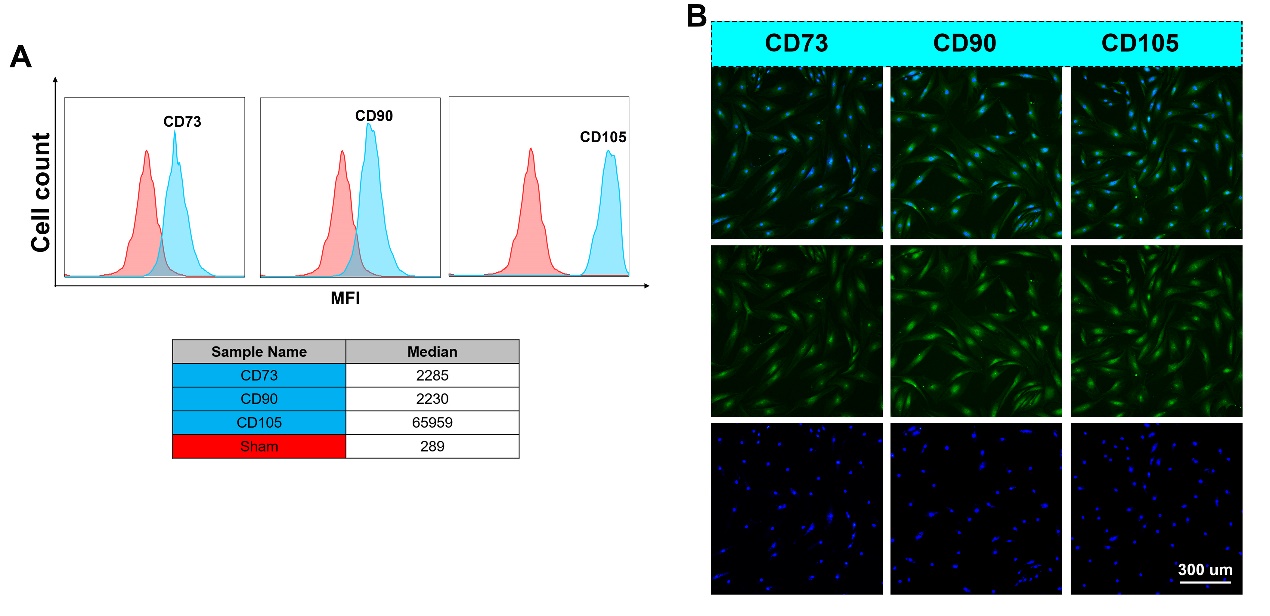


**Fig. S1** Phenotypical characterization of UCB-MSCs. **A** FCM analysis of expression profiles of cell surface markers CD73, CD90, and CD105. **B** IF staining analysis expression profiles of cell surface markers CD73, CD90, and CD105; scale bar = 300 μm.

**Table S1** Antibodis

| **Antibody** | **Company** | **Catalog number** | **Dilution** |
| --- | --- | --- | --- |
| anti-CD73 | Bioss, China | bs-4834R | 1:500 (IF); 1:100 (FCM) |
| anti-CD90 | Bioss, China | bs-20640R | 1:500 (IF); 1:100 (FCM) |
| anti-CD105 | Bioss, China | bs-0579R | 1:500 (IF); 1:100 (FCM) |
| anti-CD9 | Beyotime, China | AF1192 | 1:1000 (WB) |
| anti-TSG101 | Beyotime, China | AF8259 | 1:1000 (WB) |
| anti-CD31 | Bioss, China | bs-0468R | 1:500 (IF) |
| anti-Nestin | Bioss, China | bs-20607R | 1:500 (IF) |
| anti-Ki67 | Bioss, China | bs-23105R | 1:500 (IF) |
| Anti-α-SMA | Bioss, China | bsm-33188M | 1:500 (IF) |
| anti-Collagen I | Bioss, China | bs-10423R | 1:500 (IF) |
| anti-TGFBR1 | Beyotime, China | AF0297 | 1:300 (IF) |
| anti-TGFBR2 | Beyotime, China | AF8151 | 1:300 (IF) |

**Table S2** Primers

|  | | qRT-PCR primers |
| --- | --- | --- |
| α-SMA | F | AGCCATGTACGTAGCCATCC |
|  | R | CTCTCAGCTGTGGTGGTGAA |
| Col1a2 | F | ggtgcccctggagagaat |
|  | R | ggaccagcagacccaatg |
| TGFBR1 | F | TGCCTGCTTCTCATCGTGTT |
|  | R | TGCTTTTCTGTAGTTGGGAGT |
| TGFBR2 | F | CTGCCCATCCACTGAGACATA |
|  | R | AGCTTGGGGTCATGGCAAAC |
| GAPDH | F | TGCCCCCATGTTTGTGATG |
|  | R | TGTGGTCATGAGCCCTTCC |
| miR-21-5p | F | ACACTCCAGCTGGGTAGCTTATCAGACTGA |
|  | R | CTCAACTGGTGTCGTGGAGTCGGCAATTCAGTTGAGTCAACATC |
| miR-125b-5p | F | ACACTCCAGCTGGGTCCCTGAGACCCTAAC |
|  | R | CTCAACTGGTGTCGTGGAGTCGGCAATTCAGTTGAGTCACAAGT |
| miR-100-5p | F | ACACTCCAGCTGGGAACCCGTAAATCCGAA |
|  | R | CTCAACTGGTGTCGTGGAGTCGGCAATTCAGTTGAGCACAAGTT |
| miR-31-5p | F | ACACTCCAGCTGGGAGGCAAGATGCTGGCA |
|  | R | CTCAACTGGTGTCGTGGAGTCGGCAATTCAGTTGAGCAGCTATG |
| miR-let-7a-5p | F | ACACTCCAGCTGGGUGAGGTAGTAGGTTGT |
|  | R | CTCAACTGGTGTCGTGGAGTCGGCAATTCAGTTGAGAACTATAC |
| U6 | F | CTCGCTTCGGCAGCACA |
|  | R | AACGCTTCACGAATTTGCGT |
